# Multi-Filter Clustering Fusion for Feature Selection in Rotating Machinery Fault Classification

**DOI:** 10.3390/s22062192

**Published:** 2022-03-11

**Authors:** Solichin Mochammad, Yoojeong Noh, Young-Jin Kang, Sunhwa Park, Jangwoo Lee, Simon Chin

**Affiliations:** 1 School of Mechanical Engineering, Pusan National University, Busan 46241, Korea; msolichin1989@gmail.com; 2 Department of Mechanical Engineering, Institut Teknologi Sepuluh Nopember, Surabaya 60111, Indonesia; 3 Research Institute of Mechanical Technology, Pusan National University, Busan 46241, Korea; zmanx@pusan.ac.kr; 4 H&A Research Center, LG Electronics, Changwon 51554, Korea; sunhwa1124.park@lge.com (S.P.); jonathan.lee@lge.com (J.L.); simon.chin@lge.com (S.C.)

**Keywords:** feature selection, rotating machinery, fault classification, fusion, multi-filter, clustering

## Abstract

In the fault classification process, filter methods that sequentially remove unnecessary features have long been studied. However, the existing filter methods do not have guidelines on which, and how many, features are needed. This study developed a multi-filter clustering fusion (MFCF) technique, to effectively and efficiently select features. In the MFCF process, a multi-filter method combining existing filter methods is first applied for feature clustering; then, key features are automatically selected. The union of key features is utilized to find all potentially important features, and an exhaustive search is used to obtain the best combination of selected features to maximize the accuracy of the classification model. In the rotating machinery examples, fault classification models using MFCF were generated to classify normal and abnormal conditions of rotational machinery. The obtained results demonstrated that classification models using MFCF provide good accuracy, efficiency, and robustness in the fault classification of rotational machinery.

## 1. Introduction

Rotating machinery plays a crucial role in the systems and processes of industrial applications, such as manufacturing systems, transportation, home appliances, and power systems [1,2]. Since rotating machinery generally operates continuously at high speeds and with high power [3], interruption of the related processes could threaten safety and result in massive economic loss [4,5]. Therefore, fault diagnosis of rotating machinery is essential to prevent critical failures that would cause a system to shut down.

The fault diagnosis of rotating machine is performed by detecting outliers that may occur due to faults in the monitored data. Traditional fault detection methods have mainly used thresholds set based on domain knowledge. However, recently, many fault detection methods have detected faults by learning monitored data, using machine learning/deep learning technology. Classifying normal or abnormal conditions is performed with binary classification models, and multi-class classification models are used to detect different combinations of faults. These fault classification models help people make decisions and predict the occurrence of more severe failures in parts or machines in advance.

The most detectable signs of failures in rotating machinery are vibrations and noise from abnormal conditions. However, since noise generated under abnormal conditions is often difficult to distinguish from noise generated in external environments, vibration data are more frequently used to diagnose failures in rotating machinery. In particular, accelerometers are frequently used to measure vibration data, and various fault diagnostic methods have been developed using vibration information [6,7]. Vibration data of rotating machinery measured over time are further amplified by periodic operation of the machinery at certain frequencies. Thus, the characteristics (features) of signals, in both time and frequency domains, can be important measurements to distinguish between normal and abnormal conditions of rotating machinery. Such features are used to create fault classification models that distinguish between normal and abnormal states or different failure modes.

Selection of appropriate features (key to successfully building classification models) has been studied in recent years [8,9]. When extracting features for use as input features for classification models, it is important to use features that are highly relevant to classification, and to eliminate redundant or unnecessary features [10]. This is because learning from the data to generate a model takes a long time, and the complexity of the model increases; but the accuracy can decrease as the number of unnecessary features increases. Therefore, research on effective and accurate feature selection needs to be carried out to improve the efficiency and accuracy of fault classification models [11,12].

Various feature selection methods have been proposed for fault diagnosis in rotating machinery. In particular, since the number and type of features derived from both time and frequency domains can vary, feature selection is very important for obtaining accurate classification models. Therefore, many studies have recently been conducted to optimize the combination of features with the highest classification accuracy in feature selection. An optimal combination of features has been derived using filter methods such as relief, chi-square, and information gain [13], or by using Pareto optimization [14] or a binary particle swarm optimization method [15] after using a filter method. A wrapper-based embedded method, using a support vector machine (SVM) [16], and a method of deriving optimal features using the sensitivity of features [17] or a genetic algorithm [18] have been proposed. Although these methods have shown results in improving classification accuracy, each method has been applied to specific classification problems, which is insufficient to show the universality and robustness of the proposed methods.

Some studies have proposed effective fault diagnosis methods with several components, by extracting several features based on deep learning models using multivariate sampling data [19]. However, because these methods use a backpropagation training process, they is time-consuming and can have unsatisfactory performance when dealing with high-dimensional data. In another study, a universal domain adaptation method was proposed, to enhance the generalization ability of a data-driven model for fault diagnosis [20]. The fault diagnosis results of roller bearings showed that the proposed method yielded the best performance compared with other neural network methods. However, the study assumed that the balanced data were available in the training process. Thus, it might not be applicable to unbalanced data, which often occur in real industry applications.

This study proposes a fault classification model for rotating machinery, which is combined with a notable feature selection method, as follows: (1) Multi-filter clustering fusion (MFCF) was developed to provide an adaptive threshold capable of determining the total number of relevant features through hierarchical clustering. (2) An exhaustive search of the wrapper method was used to find the best feature sets maximizing classification accuracy. (2) The performance of the proposed method was validated in four rotating machinery cases with different operating processes, fault modes, and numbers of datasets. (3) The selected features were used to train and test several classifiers, including the SVM, k-nearest neighbors (KNN), and multilayer perceptron (MLP), to ensure that the final selected features are compatible with all classifiers. Finally, the proposed method was shown to have high accuracy, robustness, efficiency, and generalizability in fault classification for rotating machinery through multi-domain feature extraction and multi-filter fusion.

## 2. Related Methods

### 2.1. Feature Selection Methods

Feature selection can be divided into wrapper, hybrid, and embedded methods. Filter methods include methods determining the ranks of features by evaluating close relationships or similarity of features, based on information theory and statistics [17]. They evaluate the relative importance of features, but there is no absolute criterion for selecting them [21], so it is difficult to distinguish between necessary and unnecessary features [22]. Users need to arbitrarily determine the number of features, or select features according to a user-specified percentage [10], making it difficult to clearly conclude that certain filter methods are superior to others [23]. Therefore, while filter methods can efficiently remove unnecessary features based on importance, there is no guideline for selecting important features.

The most commonly used filter methods include chi-square (CS), the extra trees classifier (ETC), and a correlation matrix (CM). The CS method provides a ranking of features based on an independence test of two events using $\chi^{2}$ values. The ETC uses entropy values to measure the probability of the same class by aggregating the learning ensemble, and a CM measures the similarity between two features, with a final coefficient of the degree of linear correlation, as shown in Equations (1)–(3):

(1) $$\chi^{2}=\frac{{\left(O_{i}-E_{i}\right)}^{2}}{E_{i}}$$

where $O_{i}$ is the observed feature data, and $E_{i}$ is the expected feature data,

(2) $$\mathrm{Entropy}\;\left(E\right)=-\sum_{i=1}^{c}p_{i}\log_{2}\left(p_{i}\right)$$

where c is the number of group labels, and $p_{i}$ is the proportion of feature values associated with group i, and

(3) $$r=\frac{\sum \left(X_{i}-\bar{X}\right)\left(Y_{i}-\bar{Y}\right)}{\sqrt{\sum {\left(X_{i}-\bar{X}\right)}^{2}}\sqrt{\sum {\left(Y_{i}-\bar{Y}\right)}^{2}}}$$

where $X_{i}$ and $Y_{i}$ are feature observation data, and $\bar{X}$ and $\bar{Y}$ are the mean values of the two features, *X* and *Y*.

However, each method has different measures to evaluate feature importance, so they can yield different rankings of features. Therefore, for effective and robust feature selection, the method of extracting and combining key features from each feature selection method with different characteristics becomes an important issue, in finally deriving the best feature set.

The wrapper method determines the types and number of features based on the accuracy of classification models. All possible feature combinations are used as input features in the classification model, so the feature combination with the highest classification accuracy is chosen as the final feature set [4]. The wrapper method, unlike the filter method, provides an optimal combination of features, but requires a long computational time, because it generates a classification model for every combination of features [24,25]. Among the wrapper methods, an exhaustive search enables accurate and robust feature selection by simultaneously evaluating all combinations of features, instead of gradually adding or removing features. Exhaustive search is inefficient, compared to the other methods if all existing features are used without removing unimportant features. However, if the filter method is applied first, and the number of key features derived from the filter method is small, then an exhaustive search can find optimum features more effectively and efficiently. The hybrid method is a combination of filter and wrapper methods, to improve the shortcomings in each one. For example, after unnecessary features are removed using the filter method, the wrapper method is applied to only find the best feature set from the reduced features, resulting in a significant reduction in computational time [26,27]. While hybrid methods reduce the computational time needed with wrapper methods, they still need to select the appropriate number of features in the filter process.

### 2.2. Classifiers

Various classifiers can be applied to build classification models for the diagnostic needs of rotating machinery [28,29,30]. MLP with a neural network structure, the SVM with a decision boundary, and the distance-based KNN model are widely used classifiers. Since classifiers may have very different performances, depending on their characteristics, this study attempts to verify performance through a combination of the proposed feature selection methods, with the above three representative classifiers.

The SVM solves linear and nonlinear classification problems by finding hyperplanes that maximize the distance between groups, by learning from training data and determining the kernel type, such as linear, polynomial, or radial basal plane [31,32]. The SVM classifier is formulated as follows:

(4) $$f\left(x\right)=w^{T}\emptyset \left(x\right)+b$$

where w is the vector of the weight, b is the bias for optimizing the hyperplane, and $\emptyset \left(x\right)$ is the mapping function of the kernel. The vector of weight w can be known by minimizing it:

(5) $$\mathrm{Minimize}\;w^{T}w+C\underset{i}{\sum }\xi_{i}$$

where *C* is the penalty hyperparameter, and $\xi_{i}$ is a slack variable for *i* = 1, 2, …, *N*, with N as the number of data samples.

KNN is a type of supervised learning that can be used as a task in classification and regression. It performs classification by measuring similarity (e.g., distance functions) between data points [32]. Euclidean distance is often used as the distance metric, as follows:

(6) $$\mathrm{Dist}\left(X,Y\right)=\sqrt{\overset{n}{\underset{i=1}{\sum }}{\left(x_{i}-y_{i}\right)}^{2}}$$

where $x_{i}$ and $y_{i}$ are the coordinate values of the sample for *X* and *Y* as two data points, and *n* is the dimension of the data points. KNN attempts to find the distance between the query and all sample data. After that, it specifies the number of samples (*k*) closest to the query, and then, the most frequent label is selected.

MLP is an algorithm in machine learning that works with feed-forward neural networks. It has a structure consisting of an input layer, multiple hidden layers, and an output layer. MLP is famous for being able to solve complex problems, because of its outstanding performance in building classifications [33,34]. In simple terms, the MLP output function is expressed as

(7) $$y=g\left(W^{T}x+b\right)$$

where **x** is the input variable in vector form, y is the output; $g\left(\cdot \right)$ is the activation function of the nodes, W is the weight matrix linked to the input layer and hidden layer, and b is the bias vector of hidden layer nodes. Each component of the input layer, multiple hidden layers, and output layers can be assigned according to the level of complexity in the problem.

## 3. Proposed Method

**Figure 1:**
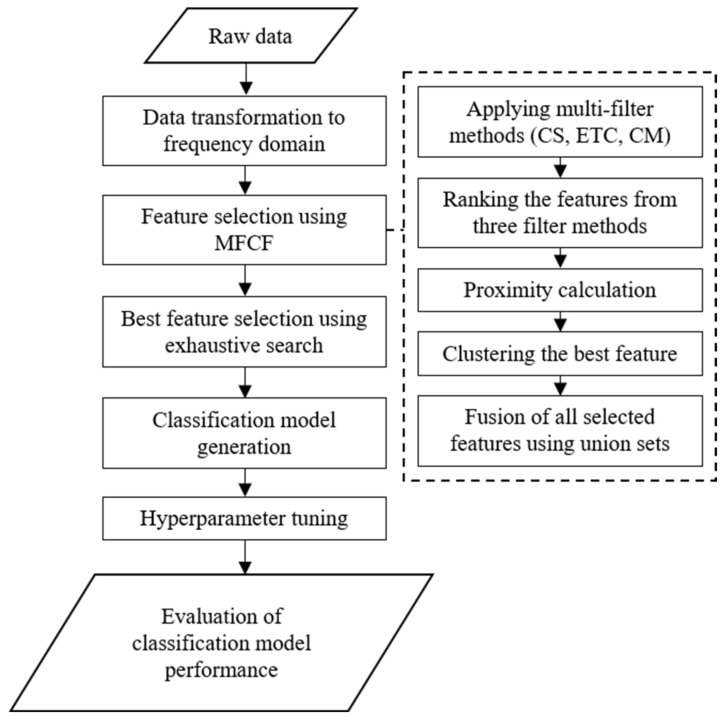
Flow chart of the proposed method.

Each filter method described in Section 2.1 can select different features depending on the type of features and the characteristics of the data, so it is important to systematically and effectively select the most important features that affect classification performance. The proposed MFCF feature selection focuses on how to cut off unnecessary features adaptively from the candidate feature sets and find the best feature combination in an efficient and systematic way. For this, the raw data are first used to extract features from time and frequency domains through fast Fourier transform (FFT), as shown in Figure 1.

MFCF is used to extract candidate feature sets using multiple filter methods and feature clustering, and an exhaustive search is used to select the optimal feature set that maximizes classification accuracy. The selected features are used to generate fault classification models (such as SVM, KNN, and MLP) where hyperparameters of the three models are optimized using a grid search. The performance of the proposed method is evaluated in terms of accuracy, efficiency, stability, and robustness. Accuracy and efficiency are evaluated using measures such as the percentage of the correct predictions and computational time, respectively. Stability is estimated from changes in both accuracy and efficiency when the method is applied to training and testing the data. Robustness is measured through variation of accuracy values, through cross validation.

### 3.1. Fusion Multi-Filter Feature Selection

Before MFCF is applied, all features from the time and frequency domains first need to be defined. In order to determine the statistical characteristics of the measured data in the time and frequency domains, 12 features were extracted from each domain, including absolute mean (abs_mean), peak-to-peak (ptp), kurtosis (kur), skewness (skew), root mean square (rms), etc.; 25%, 50%, and 75% are the 25th, 50th, and 75th percentile values, respectively. The 24 features were evaluated with CS, ETC, and CM methods. The numbering for the 72 features is in Table 1.

Referring to Table 1, feature numbering can be expressed as $F_{Comb}=\left[\begin{array}{ccc} F_{0} & F_{1}, & \begin{array}{cc} \ldots & F_{N} \end{array} \end{array}\right]$ for *k* = 0, 1, …, 71, where *k* is the list of feature numbers, and *N* is the total number. Then the term is redefined as follows: $F_{\mathrm{Comb}}=\left[\begin{array}{ccc} F_{\mathrm{CS}} & F_{\mathrm{ETC}} & F_{\mathrm{CM}} \end{array}\right]$ where $F_{\mathrm{CS}}=\left\{\begin{array}{cc} F_{f_{\mathrm{CS}}} & F_{t_{\mathrm{CS}}} \end{array}\right\}$, $F_{\mathrm{ETC}}=\left\{\begin{array}{cc} F_{f\_\mathrm{ETC}} & F_{t\_\mathrm{ETC}} \end{array}\right\}$, and $F_{\mathrm{CM}}=\left\{\begin{array}{cc} F_{f\_\mathrm{CM}} & F_{t\_\mathrm{CM}} \end{array}\right\}$. 

Clustering of the 72 features should be performed to classify them into important features and unimportant features, to be used for classification based on the feature importance measures from each filter method. For this, all feature values are normalized, and the distances between two feature values are calculated using Euclidean distance for all features, as shown in Equation (8):

(8) $$d_{ij}=d\left(F_{i},F_{j}\right)=\sqrt{\sum_{k=0}^{N}{\left(F_{i,k}-F_{j,k}\right)}^{2}}$$

where $d_{ij}$ is the distance between feature i and feature j, and N denotes the amount of data, including all feature values. Using hierarchical clustering, the distances between features are repeatedly calculated, and features with small or large distances are combined into one of two clusters: selected features or removed features. Using the Euclidean distance between two features in Equation (8), a pairwise distance matrix to find cluster A with selected features and cluster B with removed features can be defined as follows:

(9) $$d_{AB}=\left\lceil \begin{array}{cccc} 0 & d_{01} & \ldots & d_{0N}\\ d_{10} & 0 & \ldots & \ldots \\ \ldots & \ldots & 0 & \ldots \\ d_{N0} & \ldots & \ldots & 0 \end{array}\right\rceil$$

where $d_{AB}$ is a proximity matrix for measuring the distances between features. Features with short distances are clustered based on $\mathrm{Min}\;\left\{d\left(F_{i},\;F_{j}\right)\;\right\}$, and then, the proximity matrix is expressed as $d_{AB}=⎡\begin{array}{cc} \left\{d_{A}\right\} & \left\{d_{B}\right\} \end{array}⎤$. In other words, features with high proximity values are clustered into one group, whereas features with low proximity values are clustered into the other. This agglomerative clustering is repeated by building a new matrix, until the last matrix consists of only two large clusters, separating one important feature group and one unimportant feature group.

$d_{AB}=⎡\begin{array}{cc} \left\{d_{A}\right\} & \left\{d_{B}\right\} \end{array}⎤$ represents the final proximity result capable of building cluster *A* containing the feature set $\left(d_{A}\right)$, with high-ranking values from evaluating important features obtained from each single-filter method. On the other hand, cluster *B*, containing the unimportant feature set ($d_{B})$, which is far from cluster *A*, is discarded.

This feature selection is unsupervised learning, in which the algorithm automatically searches for important features by using Ward’s method, through error sum of squares (ESS) and calculating the loss associated with each cluster. The ESS is computed, to measure the distance between two clusters of important and unimportant features of multi-filter scoring, which is called the linkage function. Ward’s linkage function is known to be the most suitable method to quantify a good group based on the variance of the clusters. The target of the linkage search is to minimize the increment of the ESS at each step, to find the minimum information loss. This algorithm works by fusing two clusters as the mean vector, and it then calculates the ESS from each cluster, namely the selected feature cluster and the discarded feature cluster. The following equations define the ESS in Equation (10) and the linkage between clusters *A* and *B*, *D(A,B)*, in Equation (11):

(10) $$\mathrm{ESS}\left(d_{A}\right)=\overset{Ta}{\underset{t=1}{\sum }}{\left|Va_{i}-\frac{1}{Ta}\;\overset{Ta}{\underset{j=1}{\sum }}Va_{j}\right|}^{2}$$

(11) $$D\left(A,B\right)=\mathrm{ESS}\left(d_{AB}\right)-\left[\mathrm{ESS}\left(d_{A}\right)+\mathrm{ESS}\left(d_{B}\right)\right]$$

where Va is the value of each feature, and Ta is the number of data points in cluster *A*. With the same formula as Equation (10), $\mathrm{ESS}\left(d_{AB}\right)$ and $\mathrm{ESS}\left(d_{B}\right)$ are calculated by changing the names of the variables, such as Va to Vb as a feature value in cluster *B*, and Vab as a feature value in the combined cluster resulting from cluster fusion. Then, cluster *A* consists of several features that may be the same, due to feature selection by clustering based on all members. To optimize the combination of all potential features, after constructing a union of features selected with each filter method, redundant features are removed, and features are sorted according to importance:

(12) $$\mathrm{cluster}\_A=\left[\begin{array}{ccc} F_{CS\_new} & F_{ETC\_new} & F_{CM\_new} \end{array}\right]$$

(13) $$F_{CS{\_}_{new}}=\left\{F_{CS_{p}}|p=1,\;2,3\;,\ldots ,\;P;\;F_{CS_{p}}\in F_{CS}\right\}$$

(14) $$F_{ETC{\_}_{new}}=\left\{F_{ETC_{q}}|q=1,\;2,3\;,\ldots ,\;Q;\;F_{ETCq}\in F_{ETC}\right\}$$

(15) $$F_{CM{\_}_{new}}=\left\{F_{CM_{r}}|r=1,\;2,3\;,\ldots ,\;R;\;F_{CMr}\in F_{CM}\right\}$$

where $F_{CS{\_}_{new}\;}$, $F_{ETC{\_}_{new}}$, and $F_{CM{\_}_{new}}$ are feature sets consisting of P, Q, and R features selected using CS, ETC, and CM filter methods, respectively. Multi-filter clustering fusion can be defined as follows:

(16) $$F_{fusion}=F_{CS{\_}_{new}}\cup F_{ETC_{\_}{}_{new}}\cup \;F_{CM{\_}_{new}}$$

### 3.2. Exhaustive Search Application

The next step in the feature selection process is to derive a fusion feature set, $F_{fusion}$, that combines the selected features, by considering the accuracy of the classification model. The algorithm used to find the best combination from among all combinations of features is an exhaustive search used in the wrapper method. In this algorithm, the fusion feature has at least two to four features. The minimum number of features is determined so that the classification model can have at least two dimensions. Up to four features are used (considering the computational time), but a larger number of features can be used. The set of all features is defined as Equation (17), and the number of feature combinations is calculated using Equation (18):

(17) $$\;Y_{c}=\left\{y_{l}|l=1,\;2,3\;,\ldots ,\;C;\;y_{l}\in F_{\mathrm{fusion}}\right\}$$

(18) $$C=\sum_{s=2}^{Ss}\frac{m!}{\left(m-s\right)!s!}$$

where $Y_{c}$ is a set of all feature combinations, *C* is the number of feature set combinations, m is the length of $F_{\mathrm{fusion}}$, and *s* is the number of subset features that are combined as a target feature set, with the maximum feature combination being Ss=4.

The process of determining the combination of these feature sets is evaluated with various classifiers, such as an SVM, KNN, and MLP. Normal and abnormal data are labeled as binary levels 0 and 1, respectively, and the accuracy of the classifiers is calculated as follows:

(19) $$\mathrm{Accuracy}\left(y,\hat{y}\right)=\frac{1}{S_{\mathrm{samples}}}\;\overset{S_{\mathrm{samples}}}{\underset{l=0}{\sum }}1\left({\hat{y}}_{l}=y_{l}\right)$$

where y is the measured label values, $\hat{y}$ is the predicted label values, $S_{\mathrm{samples}}$ is the amount of data, and 1(·) denotes an indication factor. The accuracy of the classification models using all feature combinations is tested and then sorted into a set of combinations with the highest accuracy. Equation (20) is used to obtain the best combination of features based on the highest accuracy value:

(20) $$Y_{\mathrm{optimum}}=\max\left(\mathrm{accuracy}\left(Y_{c}\right)\right)$$

This study uses hyperparameter tuning to improve model accuracy, and uses 10-fold cross-validation to verify robustness. The grid search is the most representative tuning technique for computing the optimum hyperparameter value. Since it does not require much time for a small search space, and only combines a set of hyperparameters, it is simple and easy to apply. Hyperparameter optimization stops when the objective function of the hyperparameters, such as accuracy, reaches its highest value. Then, 10-fold cross-validation is performed on the generated models (based on the optimized parameters from using a grid search) and is repeated 10 times, while changing the test dataset. The accuracy of the classification model is evaluated by calculating the average accuracy for each test dataset.

## 4. Case Studies

**Figure 2:**
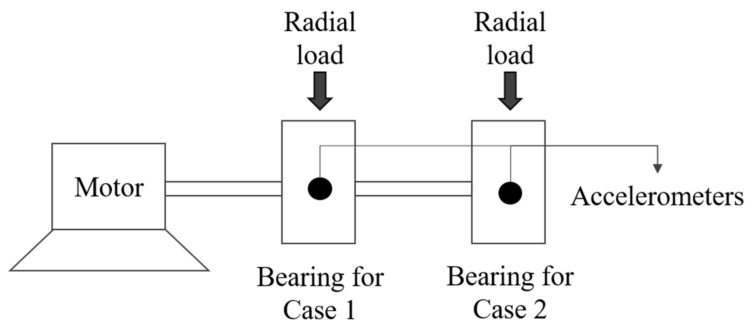
Schematic of experimental setup in cases 1 and 2.

**Figure 3:**
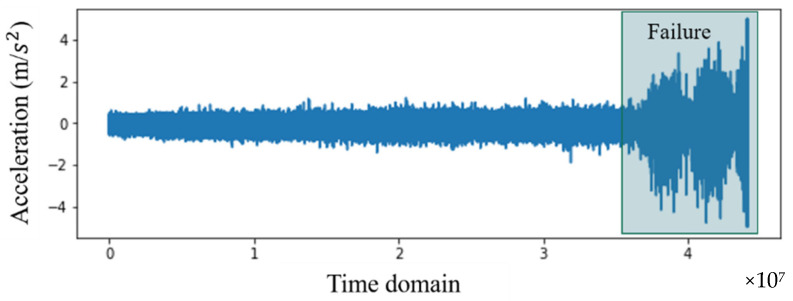
Raw data for case 1.

**Figure 4:**
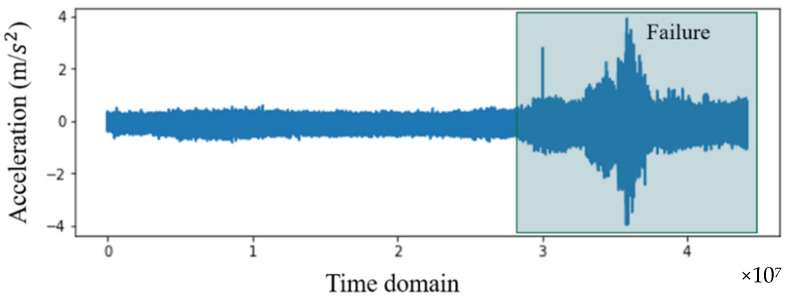
Raw data for case 2.

**Figure 5:**
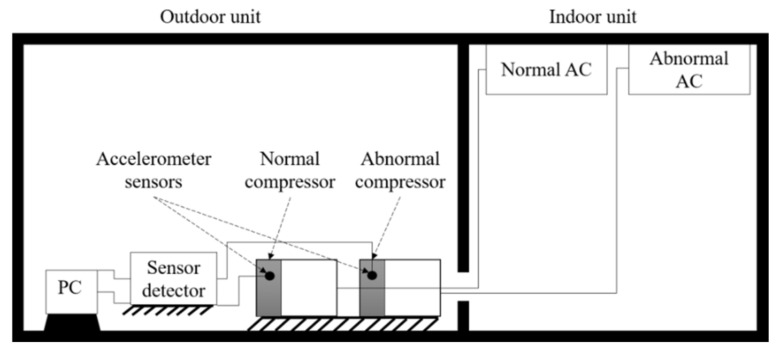
Schematic of the experiment setup in case 3 and case 4 [38].

From four examples of failures or faults that occur in different types of rotating machinery, data were collected from experiments. Cases 1 and 2 contain experimental bearing data from NASA repositories collected from the Intelligent Maintenance System Center (IMS) [35]. Cases 3 and 4 are vibration data collected from air conditioning compressors. The experiment settings in Examples 1 through 4 are shown in Figure 2, Figure 3, Figure 4 and Figure 5, and details are described in Section 4.1 and Section 4.2.

### 4.1. Data Collection

Cases 1 and 2 are rotating machinery problems that occurred with Rexnord ZA-2115 double row bearings installed on a shaft, as illustrated in Figure 2. The rotation speed of the shaft remained constant at 2000 rpm under a radial load of 6000 lbs. The bearings operated while being lubricated (so it is considered non-dry), and failure occurred after more than 100 million revolutions.

The bearing vibrations were measured using an accelerometer, recording 20,480 points at a sampling rate of 20 kHz. The data for cases 1 and 2 were recorded at 10-min intervals, and were measured 2155 times over five weeks. Cases 1 and 2 had different causes of bearing failure; case 1 had defects in the inner race, and case 2 had defects in the roller. Figure 2 shows an experiment schematic for cases 1 and 2.

Figure 3 and Figure 4 are vibration data for cases 1 and 2, measured over a five-week period. The data points along the *X*-axis are the time index at 10-min intervals. Figure 3 shows that the failure in case 1 occurred around the end of operations, at the 1789th measurement of the 2155 measurements. Therefore, data from 1789 measurements can be classified as normal, and 366 can be classified as abnormal. On the other hand, Figure 4 shows that the failure in case 2 occurred earlier than in case 1, during the 1434th measurement. Therefore, in case 2, there were 1434 normal measurements, and 471 abnormal measurements. The normal and abnormal data were divided based on the history of the vibrations, as shown in Figure 3 and Figure 4, and based on the threshold for kurtosis (a feature mainly used in fault classification). In addition, data segmentation was verified through an operation indicating the time when bearing abnormalities occurred [36,37].

In cases 3 and 4, vibration data collected from an air conditioner compressor were used to apply the proposed method to fault data with various characteristics. The faults in these two cases were caused by two different failure modes (a mechanical defect, and lack of refrigerant inside the compressor). The machine used in cases 3 and 4 was a twin rotary compressor with low vibration and a 180° phase difference when rotating the shaft. Figure 5 shows a schematic for cases 3 and 4.

The experiment was conducted in two different rooms, an outdoor unit and an indoor unit, to simulate actual conditions for using air conditioners. In cases 3 and 4, an accelerometer measured the vibrations in the compressors shown in Figure 5. Details of the experiment variables in cases 3 and 4 are shown in Table 2 and Table 3, respectively.

In case 3, both normal and abnormal compressors operated, consisting of six electric expansion valve (EEV) variables × three fan-speed variables × four frequency variables × two conditions = 144 measurements. To improve the accuracy of the classification model, the data were partitioned into 50 intervals of the compressor cycle, increasing to 7200 measurements in total [39]. Case 4 collected normal and abnormal data at seven frequencies, where each variable was repeated three times. A state in which the refrigerant is charged at the 100% level is considered normal, and a state in which the refrigerant level is 50–90% is considered abnormal. This indicates that the refrigerant charge gradually declined, due to continuous operation of the air conditioner. Therefore, there are 39 normal measurements and 216 abnormal measurements.

### 4.2. Feature Extraction and Selection

The data in this study constitute the time domain and the frequency domain. The data measured in the time domain are acceleration. The data in the frequency domain were obtained by transforming time domain data using fast Fourier transform (FFT), as follows:

(21) $$f_{k}=\overset{L}{\underset{r=0}{\sum }}x_{r}e^{-2\pi j\frac{kr}{L\;\;}}$$

where *L* is the length of the data sequence of $x_{r}$ as input time domain, and k=0, 1, …L. As described in Table 1, 12 features were extracted from each domain, for a total of 24 features. Thus, two input feature matrices were formed into time-domain and frequency-domain combinations: $F_{t}=\left[\begin{array}{ccc} F1_{t} & \ldots & F12_{t} \end{array}\right]$, $F_{f}=\left[\begin{array}{ccc} F1_{f} & \ldots & F12_{f} \end{array}\right],$ and $F=\left[\begin{array}{cc} F_{t} & F_{f} \end{array}\right]$.

**Figure 6:**
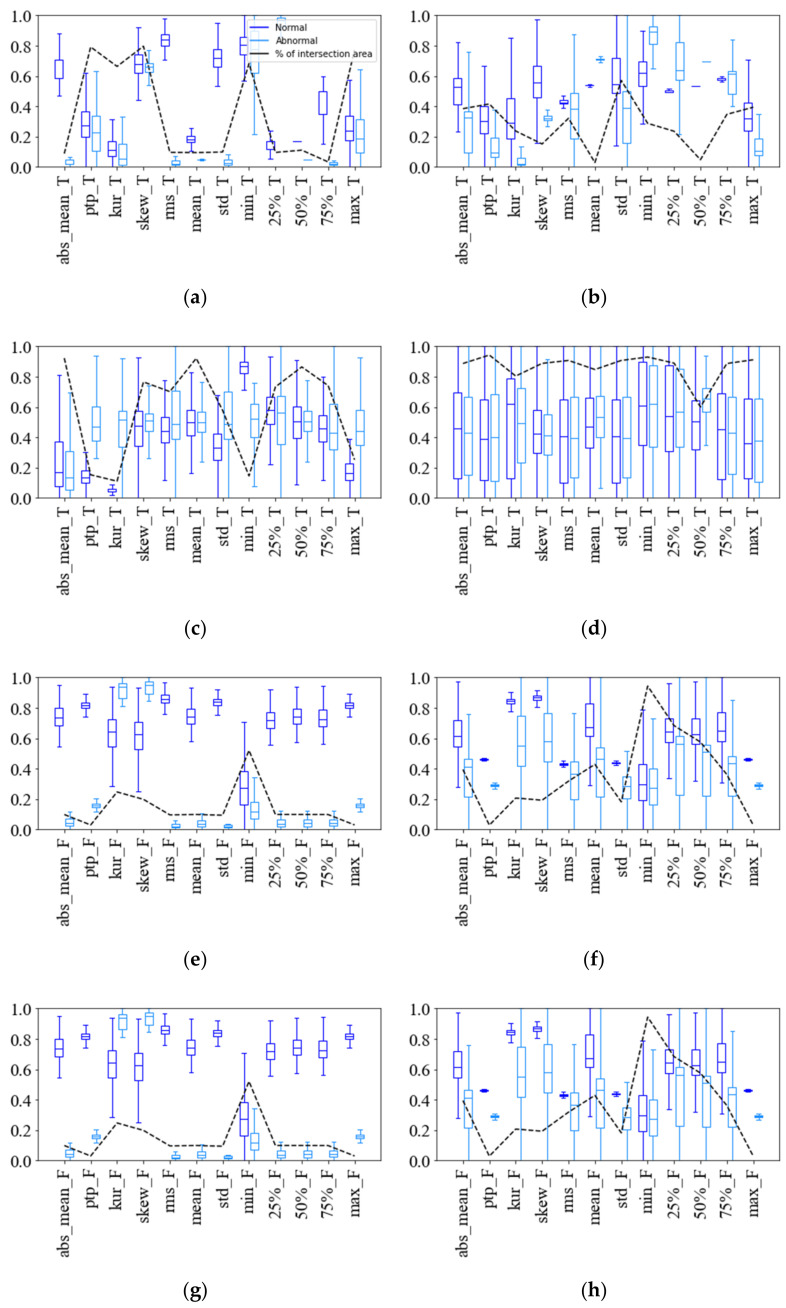
Distribution and intersection areas of normalized feature values. (**a**) Time domain features for case 1; (**b**) Time domain features for case 2; (**c**) Time domain features for case 3; (**d**) Time domain features for case 4; (**e**) Frequency domain features for case 1; (**f**) Frequency domain features for case 2; (**g**) Frequency domain features for case 3; (**h**) Frequency domain features for case 4.

After normalizing using a min–max scaler method with the data in both domains, box plots of the data for each feature can be obtained for the four cases, as shown in Figure 6, where the intersection refers to the overlapped normal and abnormal data distributions. The intersection of normal and abnormal data distributions is used to show whether each feature sufficiently distinguishes between normal and abnormal data. The smaller the intersection area, the more easily the corresponding feature classifies normal and abnormal data; the larger the intersection area, the more difficult the classification is. Thus, the intersection area can be used as an initial estimate of whether the feature is easy or difficult to classify into normal and abnormal conditions.

As seen in Figure 6, the intersection areas vary greatly, depending on the feature types and cases. Some features have a small intersection area, which means they can clearly distinguish between normal and abnormal data, while others are not useful for fault classification. Case 1 has the smallest intersection area, indicating that failure classification is easiest. However, a large number of key features can be selected, so the number of features needs to be reduced to improve fault classification accuracy and decrease the computational time. On the other hand, the distributions of normal and abnormal data for most features are not clearly distinguished in case 3, and their intersection areas are close to 1 in both time and frequency domains, making it very difficult to derive important features. Case 4 shows that some features in the time domain are valid, but most features in the frequency domain are invalid. The results from case 4 confirm that using multiple domains rather than a single domain helps improve the accuracy of fault classification. In summary, each case had a different number and type of features extracted, due to different causes of failure and the different data characteristics. Therefore, it is necessary to correctly select the type and number of features suitable in each case.

**Figure 7:**
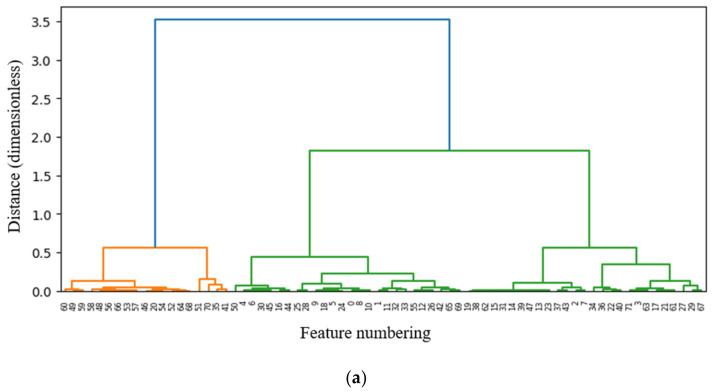

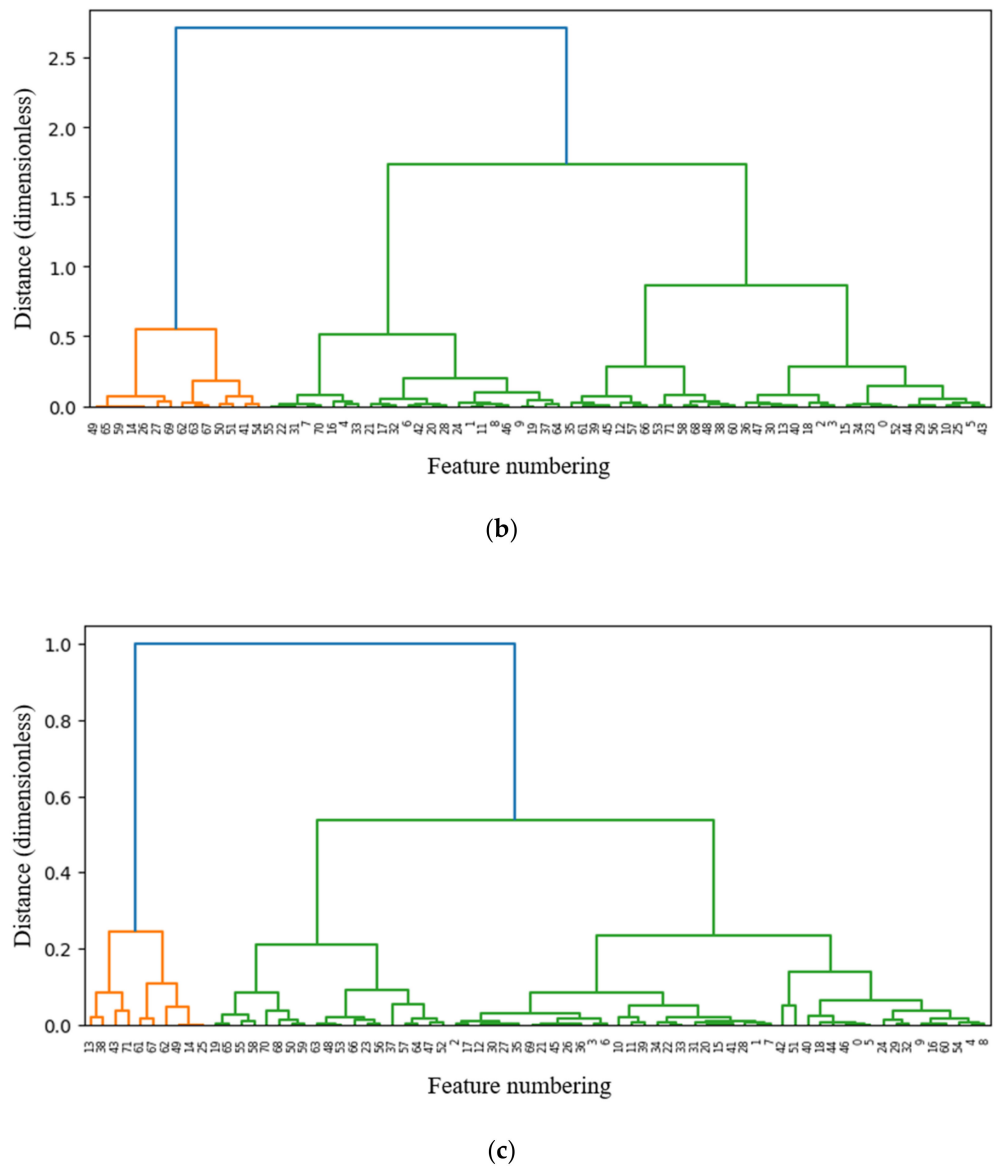

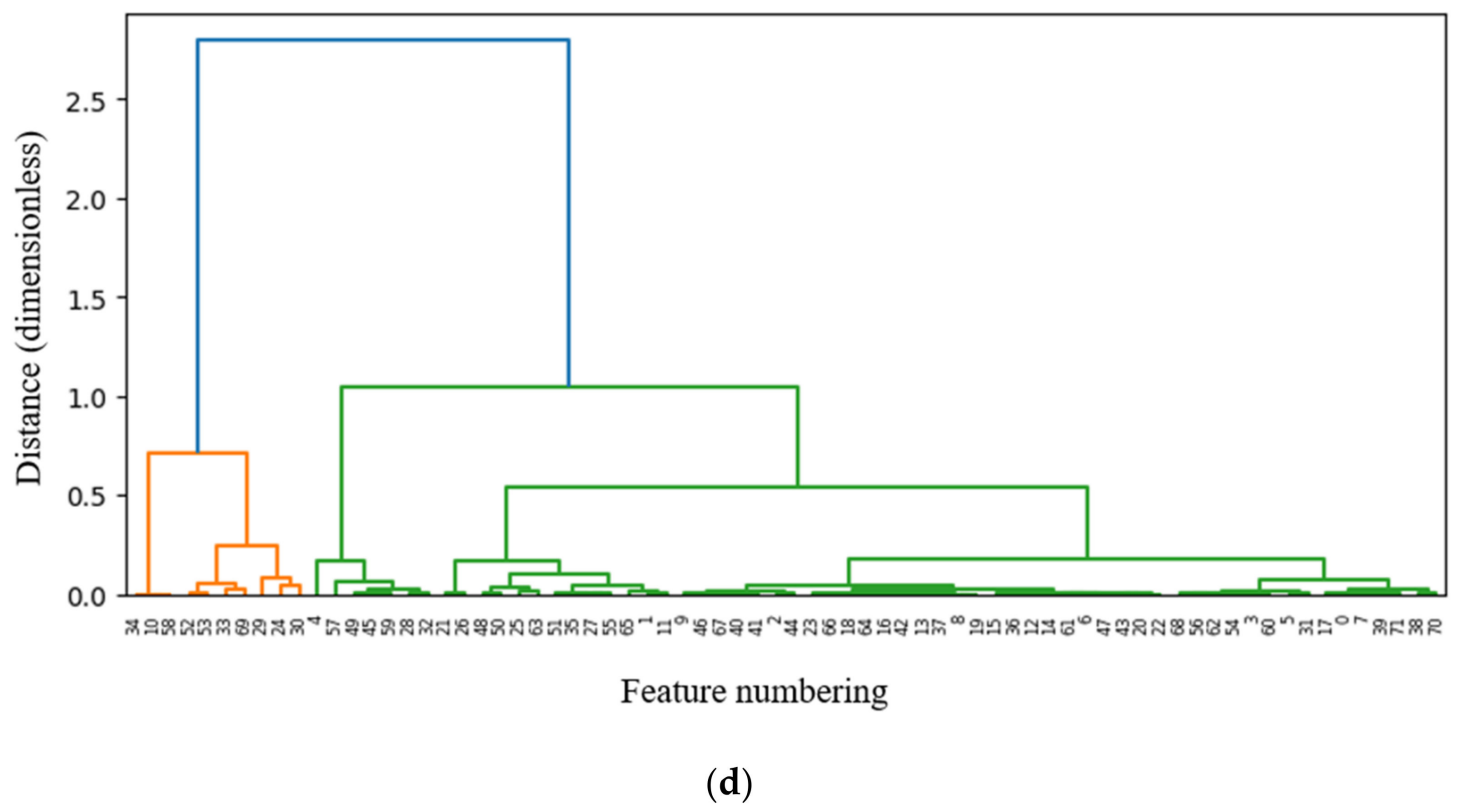
Multi-filter clustering. (**a**) case 1; (**b**); case 2; (**c**) case 3; (**d**) case 4.

Figure 7 shows a dendrogram of the results from hierarchical clustering obtained for all features by applying MFCF in cases 1 through 4. The dendrogram represents the hierarchical relationship between the clusters, where the *X*-axis represents feature numbering (see Table 1) listed by importance, and the *Y*-axis represents the proximity of the Euclidean distance between two features. The features can mainly be clustered into two groups (orange lines and green lines). The orange lines include the main features with high importance and proximity, and the green lines include features that need to be deleted based on the three filtering methods. Cases 1 through 4 have data with different characteristics, so the types and numbers of selected features are different in all cases.

In order to remove the same features at the clustering stage, the total number of features selected before sorting via fusion of the three filter methods are 19, 14, 9, and 10 for Cases 1, 2, 3, and 4, respectively. As expected from the results in Figure 6, case 1 contains the largest number of features classified as main features. On the other hand, cases 3 and 4 have a smaller number of main features for classification, so the number of selected features is less than in cases 1 and 2. Hierarchical clustering allows users to easily derive valid features, by dividing all features into necessary and unnecessary sets. However, the number of clustered features is still large, so it needs to be further reduced. Details of the feature reduction process at each stage (multi-filter clustering, fusion, and the proposed method) are shown in Table 4.

Table 4 shows the features selected in each case with different subsets at each MFCF stage, where the three numbers in the last column of the final set indicate the number of features used in SVM, KNN, and MLP, respectively. Since data in each case are measured from different rotatory machines with different failure modes, each case has different numbers and types of important features extracted from the different domains. For example, in case 1, mean_T was used as the input feature in the three classifiers. The acceleration time series data have a sine or cosine curve with almost the same amplitude, so mean_T tends to have a constant value. However, since the abnormal data differ from the average values of acceleration of normal data, mean_T may be an important feature for fault classification. In case 2, skew_T was used as an input feature, because skewness measures the asymmetry of the probability density function of the vibration signals. In case 3, kur_T, ptp_F, and min_T were used as common input features for the three classifiers, where kur_T and ptp_F indicate the degree of flatness of the probability density function near the center and the peak value of the signals, respectively. They are often used to measure the strength of signals, due to failure of rotating machinery. Furthermore, min_T shows that the normal compressor condition had a low minimum value for acceleration response, compared to the minimum value under abnormal compressor conditions. In case 3, the intersection areas for many of the features are high, i.e., there are few important features except those three features shown in Figure 6, and they were used as input features in the fault classification models. Accordingly, the proposed method can be used more effectively in a problem that is difficult to classify. The most frequently selected features in case 4 are mean_F and abs_mean_F. The amplitude of the vibration signal of rotatory machinery is particularly useful for distinguishing between a normal state and an abnormal state in the frequency domain. Therefore, mean_F and ABS_mean_F functions were selected as common main features. After the training process, to build the classifiers for the finally selected features in all cases through the exhaustive search, two to four feature combinations were derived.

Using the finally selected features, fault classification models were generated using SVM, KNN, and MLP. Table 5 shows the accuracy and calculation times of the three single-filter methods and the proposed MFCF. The proposed method was compared with the three single-filter methods using the top three features, because it can present a good comparison by selecting the most useful features from the top three, and it can control the computational time. The accuracy of the proposed method was 1.0 (100%) for all classifiers in cases 1 and 4, and cases 2 and 3 had an average accuracy of 0.99 for all classifiers. In terms of efficiency, the proposed method consumed the least computational time compared to the others, because the exhaustive search was only performed for the selected features through MFCF, and the randomness in feature selection was low. Conversely, CS returned the lowest accuracy and required the longest running time, even though it is not much different from the other feature selection methods. In particular, the CS method yielded the lowest accuracy in case 4 (with the smallest number of samples), because it depends on sample size, and its time consumption was drastically different from the other methods, owing to the selection of classifiers. KNN was the most computationally expensive classifier. This was due to the complexity of the algorithm that stores the training data, as well as the number of iterations needed to calculate the distance between feature values. Thus, the proposed method was the most efficient, and yet had it the highest classification accuracy.

These high-accuracy and low-computational times are highly advantageous for machine learning, especially when diagnosing failures in rotating machinery with many classification difficulties. The performance of the proposed method was validated by testing several cases with different characteristics, such as the number of datasets, the types of failures, the types of experimental objects, and the variables in the data collection, as described in the previous section.

**Figure 8:**
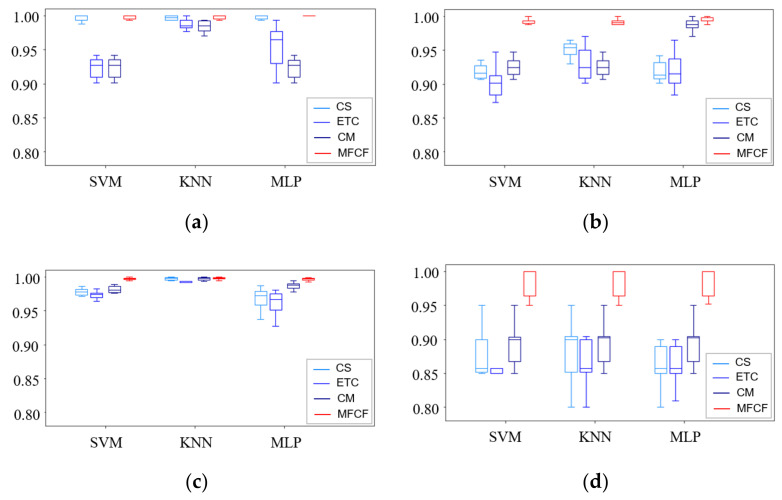
Ten-fold cross validation. (**a**) case 1; (**b**); case 2; (**c**) case 3; (**d**) case 4.

To validate the classification model, 10-fold cross-validation was carried out to determine the general applicability of the proposed method. Figure 8 shows box plots for the accuracy results from CS, ETC, CM, and the proposed MFCF. Comparing each method, CS generally had low accuracy, high variability, and varying results, depending on the classifier type. CM tended to be similar to the results from CS, indicating that accuracy varies according to the classifier. ETC often had a higher accuracy than the other filter methods, but still had a lower accuracy and higher variability than the proposed method. On the other hand, the proposed method had little variability in the results, although accuracy was close to 1.0 in cases 1 to 3, where classification is easy regardless of the classifier type. However, in case 4, we can see that the lack of data resulted in lower classification accuracy and higher variability than in the other cases, but it still showed the best accuracy in comparison with the other methods.

## 5. Conclusions

This study developed a hierarchical clustering method using multiple filters (called MFCF), to extract key features from time and frequency domains, and to maximize classification accuracy by optimizing the number and type of features using an exhaustive-search-based wrapper method. MFCF enables robust, accurate, and efficient fault classification, regardless of the type of failure classification model, especially in the fault classification of rotatory machinery, including complex failure modes and different data characteristics. To validate the proposed method, vibration data from rotating machinery with four different failure modes were used, and cross-validation results confirmed that it had the best classification performance, compared to the other filter methods. Although the proposed method in this study was used for the problem of classifying normal measurements and those with abnormalities, it will be applied in the future to problems including multi-classification and multi-domain features, to verify its general applicability to broad engineering applications. In addition, this study obtained vibration signals using only accelerometer sensors, but the proposed method will be applied to extract features of data collected using various sensors, such as chemical and temperature sensors in the future.

## Figures and Tables

**Table 1:** Feature number.

Frequency Domain Features	$F_{f\_CS}$	$F_{f\_ETC}$	$F_{f\_CM}$	Time Domain Features	$F_{t\_CS}$	$F_{t\_ETC}$	$F_{t\_CM}$
abs_mean_F	0	24	48	abs_mean_T	12	36	60
peak_m_F	1	25	49	peak_m_T	13	37	61
kur_F	2	26	50	kur_T	14	38	62
skew_F	3	27	51	skew_T	15	39	63
rms_F	4	28	52	rms_T	16	40	64
mean_F	5	29	53	mean_T	17	41	65
std_F	6	30	54	std_T	18	42	66
min_F	7	31	55	min_T	19	43	67
25%_F	8	32	56	25%_T	20	44	68
50%_F	9	33	57	50%_T	21	45	69
75%_F	10	34	58	75%_T	22	46	70
max_F	11	35	59	max_T	23	47	71

**Table 2:** Experiment variables in case 3.

Conditions	EEV	Fan Speed (rev/min)	Frequency (Hz)
Cooling	60, 120, 180, 240, 300, 360	350, 500, 700	20, 30, 40, 50
Heating	60, 120, 180, 240, 300, 360	350, 500, 700	20, 30, 40, 50

**Table 3:** Experiment variables in case 4.

Conditions	Refrigerant (%)	Frequency (Hz)
Normal	100	30~90
Abnormal	50~90	30~90

**Table 4:** Selected features at each stage.

Cases	Stages	Features	No. of Features
Case 1	Multi-filter clustering	{25%_T} ∪ {max_F, mean_T} ∪ {ptp_F, 75%_T, abs_mean_F, rms_F, mean_F, std_F, 25%_F, 50%_F, 75%_F, max_F, abs_mean_T, rms_T, std_T, 25%_T, 75%_T, skew_F}	19
	Fusion	25%_T, max_F, mean_T, ptp_F, 75%_T, abs_mean_F, rms_F, mean_F, std_F, 25%_F, 50%_F, 75%_F, abs_mean_T, rms_T, std_T, 75%_T, skew_F	17
	Final set	SVM: rms_T, 75%_T, KNN: mean_T, 75%_T, mean_F, MLP: mean_T, 75%_T, ptp_F	2,3,3
Case 2	Multi-filter clustering	{kur_T} ∪ {kur_F, skew_F, mean_T} ∪ {kur_F, skew_F, mean_T, ptp_F, std_F, max_F, kur_T, skew_T, min_T, 50%_T}	14
	Fusion	kur_T, kur_F, skew_F, mean_T, ptp_F, std_F, max_F, skew_T, min_T, 50%_T	10
	Final set	SVM: skew_F, std_F, kur_T, skew_T, KNN: kur_T, skew_F, std_F, skew_T, MLP: kur_F, max_F, skew_T, mean_T	4,4,4
Case 3	Multi-filter clustering	{ptp_T, kur_T} ∪ {kur_T, min_T} ∪ {ptp_F, ptp_T, kur_T, min_T, max_T}	9
	Fusion	ptp_T, kur_T, min_T, ptp_F, max_T	5
	Final set	SVM: ptp_T, kur_T, ptp_F, min_T, KNN: ptp_T, kur_T, ptp_F, min_T MLP: kur_T, ptp_F, min_T, max_T	4,4,4
Case 4	Multi-filter clustering	{75%_F} ∪ {75%_F, 50%_F, rms_F, abs_mean_F, std_F} ∪ {75%_F, rms_F, mean_F, 50%_T}	10
	Fusion	75%_F, 50%_F, rms_F abs_mean_F, mean_F, std_F, 50%_T	7
	Final set	SVM: abs_mean_F, rms_F, mean_F, KNN: abs_mean_F, mean_F, std_F, MLP: 75%_F, abs_mean_F, mean_F, std_F	3,3,4

**Table 5:** Accuracy and execution times with the test data.

	Methods	Case 1			Case 2			Case 3			Case 4			
		SVM	KNN	MLP	SVM	KNN	MLP	SVM	KNN	MLP	SVM	KNN	MLP	Avg.
Accuracy	CS	0.93	0.99	0.93	0.91	0.95	0.92	0.97	0.99	0.96	0.76	0.94	0.95	0.93
	ETC	0.98	0.99	0.96	0.88	0.95	0.93	0.98	0.99	0.97	0.86	0.99	0.96	0.95
	CM	0.93	0.98	0.93	0.93	0.97	0.98	0.98	0.99	0.98	0.94	0.98	0.96	0.96
	MFCF	1.0	1.0	1.0	0.99	1.0	0.99	0.99	0.99	0.99	1.0	1.0	1.0	0.99
Efficiency (sec.)	CS	3.52	114.9	54.2	6.75	104.2	47.62	90.2	659.5	80.94	0.4	30.3	11.4	100.3
	ETC	3.01	115.4	23.9	5.23	103.6	52.7	84.1	678.9	70.89	0.4	30.8	13.4	98.5
	CM	3.5	118.6	43.3	4.99	100.9	36.6	86.5	680.8	69.27	0.4	30.5	9.1	98.7
	MFCF	2.4	113.2	13.8	4.02	100.1	39.6	83.0	655.3	81.63	0.4	30.0	10.0	94.4

## Data Availability

Not applicable.
